# The influence of microclimate conditions on ozone disinfection efficacy in working places

**DOI:** 10.1007/s11356-021-15457-2

**Published:** 2021-07-27

**Authors:** Concetta Pironti, Giuseppina Moccia, Oriana Motta, Giovanni Boccia, Gianluigi Franci, Emanuela Santoro, Mario Capunzo, Francesco De Caro

**Affiliations:** 1 Department of Medicine Surgery and Dentistry “Scuola Medica Salernitana”, University of Salerno, via S. Allende 1, 84081 Baronissi, SA Italy; 2 AUO San Giovanni di Dio e Ruggi d’Aragona Hospital, via S. Leonardo, Salerno, Italy

**Keywords:** Ozone, Disinfection, Health safety, Indoor environments, Microclimate conditions

## Abstract

In recent years, the sanitization of environments, devices, and objects has become mandatory to improve human and environmental safety, in addition to individual protection and prevention measures. International studies considered ozone one of the most useful and easy sanitization methods for indoor environments, especially hospital environments that require adequate levels of disinfection. The purpose of this work was to evaluate the microclimate influence on sanitizing procedure for indoor settings with ozone, to prevent infections and ensure the safe use of the environments. The concentration of ozone was measured during sanitization treatment and estimation of microorganisms’ survival on the air and different contaminated plates after the sanitization operations were performed. The results demonstrated a significant reduction in the microbial count that always fell below the threshold value in different conditions of distance, temperature, and relative humidity.

## Introduction

In the last year, a new worldwide emergency introduced the requirement of new disinfection and sanitation procedures to optimize the quality of care and work safety in professional environments [Amato et al. 2020; Brunetti et al. 2006, 2008; Esposito et al. 2017; Fraise 1999; Gilbert and McBain 2003; Hoy 1981; Jakobsson et al. 1991; Moccia et al. 2020a; Motta et al. 2008, 2015, 2018; Pitten et al. 2003; Pironti et al. 2021; Proto et al. 2016; Sauerbrei et al. 2012]. In particular, ozone-producing devices were used as the easiest and most efficacious disinfection and sanitization method to prevent the spread of multiresistant microorganisms in hospital wards [Knobler et al. 2004; Food and drug administration 1982; Rubio-Romero et al. 2020; Moccia et al. 2020b; Sousa et al. 2011]. The high efficiency of ozone was evaluated against many microorganisms, fungi and viruses both on the surfaces and suspended in the air [Dubuis et al. 2020] and, for this reason, was also validated by many international organizations [Environmental Protection Agency 2020]. The use of ozone was associated with its properties: high oxidizing power towards all types of organic and inorganic compounds; complete inactivation of microorganisms that can be present on the surfaces but also under the surfaces of furniture; great sanitizing power for the air. However, only a few recent studies investigated the relationship between ozone concentration and microclimate conditions of different environments [Blanco et al. 2021]. Some experiments demonstrated that the ozone concentration and the relative humidity values played an important role in the efficiency of ozone and its antimicrobial effect [Grignani et al. 2021]. Hudson evaluated the effect of concentration, time of exposure, and relative humidity on 12 viruses. The results of this work showed a reduction of three orders of magnitude, with respect to the initial virus title, at a concentration of 25 ppm of ozone for 15 min exposure at >90% RH [Hudson et al. 2009]. Although this could be considered an encouraging result, the ozone concentration used in the experiment is very high and its oxidizing effects could be risky for operators and environments with degradation of several materials. The right compromise should be to find the optimal dose and time of usage sufficient to destroy microorganisms with the least degradation effects on materials and impairment to human health. Moreover, US EPA studies highlighted the correlation between human exposure and ozone-induced decreases in lung function and inflammation in healthy, exercising adults at concentrations as low as 60 ppb after 6.6 h of exposure [Environmental Protection Agency 2020]. The available epidemiologic evidence suggests detrimental health effects such as inhalation toxicity, skin corrosion and serious eye damage on exposure to even low doses of ozone [Jaffe 1967; Lee et al. 1996; Salvador et al. 2019].

Although the biocidal efficiency of ozone was investigated in different conditions, studies for its use in indoor environments (not laboratory scale) are missing, and this aspect has to be necessarily improved to avoid misleading circumstances that could cause considerable outbreaks. An efficient disinfection process with ozone could be obtained through the good distribution of gas in large areas, no stagnant regions with lower ozone concentrations and a circulating fan to ensure a uniform flow through the entire room during sanitization. Moreover, utilization of the right dose in different conditions is fundamental since low concentrations and time of exposure are unlikely to be effective in sanitization, and inefficient disinfection would represent an additional risk to people that could reduce their personal protection feeling safe [Motta et al. 2021]. The advantages of ozone over traditional disinfectants are related to low costs, easy production and use, no disinfection residues, and its capability as gas to penetrate each part of a room. Our work analyzed the effects of environmental conditions such as temperature, humidity, and the distance from the ozone-generator on the decontamination of air and surfaces in a healthcare office and a classroom occupied and unoccupied by students.

## Materials and methods

Ozone was generated in-situ using a portable commercial ozone-generating apparatus, with an average production of 1.6 ppm/h. The generator is equipped with a device that guarantees a total reduction of ozone concentration at the end of the treatment. Automatically, as described by the suppliers, at the end of the ozonation phase, the catalyzing phase begins. During this latter, the residual ozone in the air passes through the UV-C lamps, which converts ozone into oxygen eliminating any residue.

**Fig. 1:**
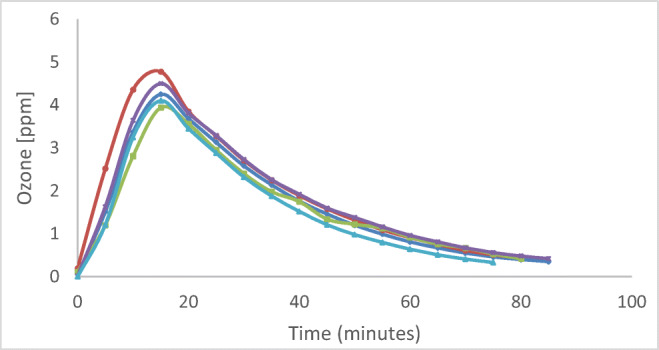
Ozone concentration in ppm evaluated at 1 m red, 2 m blue, 3m light blue, 5 m violet, 12 m green

Ozone concentration was evaluated by means of Airnova sensors, calibrated and certified by the suppliers. In Fig. 1, the curves of ozone concentration during sanitization until the disappearance after 70 min are reported. The maximum value obtained was 4.80 ppm after 20 min and the average value was 1.6 ppm for the entire ozone-generation time. The ozone concentration was measured in different points of the room in comparison with the position of the generator and the curves showed represent the measurements performed. Two different kinds of rooms were analysed: a 150 m^2^ classroom (volume of 600 m^3^ ) with and without students, and a healthcare office of about 60 m^2^, with a volume of 180 m^3^ (ozonation was always conducted in the absence of people).

The plates used for the microbial count were contaminated with representative gram-negative *E. coli* (ATCC 25922) bacteria, reconstituted from a deep-frozen stock (storage at −80°C). After lightly scratching the surface of the frozen stock by a sterile inoculating loop, bacterial cultures were suspended in Luria-Bertani liquid medium (LB) using a vortex mixer and grown in a shaking incubator at 37°C for 12 h, which lead to a cell density of approximately 1 × 10^9^ CFU/mL. Bacterial growth was verified by measuring the optical density (OD at 600 nm; OD600) of two 1:10 diluted aliquots by spectrophotometry (Thermo Spectronic, Heλios γ). The calibration showed the linear association of the optical density at 600 nm (OD600) vs CFU/mL values for the tested bacterial strain. The plates were then exposed at an average concentration of 1.6 ppm ozone for 70 min at temperatures 16, 21 and 25°C and relative humidity of 35, 45 and 55%. In each experiment, plates placed for 70 min without ozone exposure were used as controls. The plates were positioned at a different distance from the ozone generator at 1, 1.5, 2, 3, 5 and 12 m on the same level and 1 and 2 m in height.

Air samples were collected by SAS 180 S (SURFACE AIR SYSTEM monitoring instruments) system for microbiological environmental monitoring, used in combination with contact plates. The instrument was positioned one/two meters from the ozonization system, which was calibrated to start measurement after 5 min to eliminate interferences of operators in the room and sampling 1000 L of air in 6 min. The measurements were done before, during, and after ozone treatments. Microbiological analyses in the air were performed using a 24 cm^2^ Rodac (Replicate Organism Direct Agar Contact) plate with a PCA (plate count agar to total microbial count) substrate, specific for the monitoring of environment hygiene (air and surface). The plates were incubated under aerobic conditions at 30 °C for 48 h. The number of microorganisms per plate (CFU) was calculated from the number of colonies obtained on the plates containing less than 300 colonies/plate.

## Results and discussions

The influence of microclimate conditions on the efficiency of ozone as a sanitation system is shown in Tables 1–2 that summarize the results of the measurements in the office.

**Table 1:** Microbial load (CFU/plate) estimated on plates exposed at different distances and heights (results are averaged on 10 measurements)

**Distance of plates from generator (m)**	**Post treatment** **(CFU/mL)** **Floor level**	**sd**	**Post treatment** **(CFU/mL)** **1 m height**	**sd**	**Post treatment** **(CFU/mL)** **2 m height**	**sd**
1	15	±3	25	±5	23	±2
1.5	20	±2	17	±1	25	±2
2	22	±3	19	±4	26	±2
3	20	±2	23	±1	16	±2
5	17	±1	26	±1	22	±1
12	24	±1	31	±4	27	±2

**Table 2:** Microbial load (CFU/plate) estimated on plates exposed at different distances and different values of RH (results are averaged on 10 measurements)

**Distance of plates from generator (m)**	**Post treatment** **(CFU/mL)** **35% RH**	**sd**	**Post treatment** **(CFU/mL)** **45% RH**	**sd**	**Post treatment** **(CFU/mL)** **55% RH**	**sd**
1	16	±3	19	±5	15	±2
1.5	22	±2	18	±1	25	±3
2	24	±1	25	±4	26	±2
3	18	±1	25	±1	23	±2
5	21	±1	26	±3	23	±1
12	32	±3	30	±2	27	±2

The results reported in the tables show that the ozone sanitization system succeeds to eliminate about 90% of the microorganisms present on the analyzed plates in different conditions of distance, and relative humidity. Different temperatures were also tested but the results were comparable and it was not considered useful to report them.

The choice to work at low ozone concentration was related to the awareness of the dangerous effects on rubber and metals at high concentrations, due to its oxidizing power. Limited studies evaluated the progressive damage of materials and products, shortening their life with significant economic losses for industries and other activities [Lee et al. 1996; California Environmental Protection Agency 2020]. The reduction of ozone concentration is necessary to avoid consequences on materials (rubber, nylon, acetate, metals, etc.), human and environmental safety [James 1985]. For this reason, a high concentration could be used in critical environments and circumstances only for short periods. In previous studies, the total ozone dose was considered an important factor for biocidal activity and it is calculated as the product of exposure time and concentration [Dennis et al. 2020]. Tseng and Li 2008 reported that the required ozone dosage for 99% viral inactivation should be calculated as ppm × minutes (i.e. product of ozone gas concentration multiplied by duration). To inactivate (99% reduction) dsDNA(T7) virus 114 min[ppm] is required at 55% relative humidity and even lower for other viruses (37.99, 30 for ϕx174, MS2, ϕ6 respectively). In our case, we always obtained a value of 112 min [ppm] by calculating the average concentration (1.6 ppm) and the duration of exposure (70 min), very close to the value suggested by Tseng and Li to inactivate viruses. In all experiments, we obtained a significant reduction of microbial load, demonstrating the effectiveness for all environmental conditions analyzed. In fact, independently of the distance, height, temperature, and RH, we found rather low contamination on the plates compared to the control where a 6.8*10^6^ CFU/plate was observed on average. The concentration of ozone used permits obtaining a good balance between microbial load reduction and reduced damage on materials in the healthcare office.

**Fig. 2:**
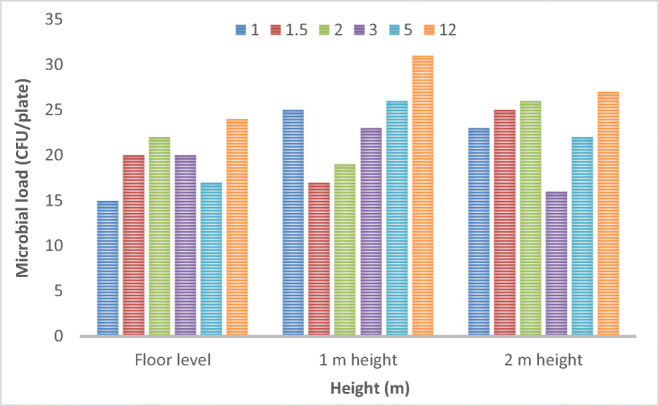
Evaluation of Microbial load estimated after ozone disinfection at different distances and heights from the ozone generator

In Fig. 2, the microbial load determined after ozone disinfection at different distances and heights is reported. It can be observed that the concentration of microorganisms decreased equally following exposure to ozone at different distances. The results are in agreement with those reported by Zucker et al. 2021 which were done in a reaction chamber equipped with a miniature table and cabinet to simulate indoor contamination.

In Fig. 1, the ozone concentration measured in various points of the room is reported, and we can observe an equal distribution of gas in the environment with the ozone concentration showing the same trend for all the positions analysed. The ozone concentration reaches a maximum value of 4.80 ppm after 20 min and the complete disappearance after 70 min. Although there is a natural ozone reduction in the air, a faster residual ozone removal can be achieved by using adsorbents and/or catalytic converters, as in this case. This is an interesting and unprecedented evidence of the ozone disinfection potential because in real indoor environments, it is possible to disinfect surfaces not typically disinfected with manually applied liquid disinfectants, such as the back of plane seats or the top of furniture. However, the correct use of ozone is related to many factors, i.e. ozone concentration, the temperature of the environment, humidity of the environment and exposure time. For these reasons, we have tested the efficiency of ozone disinfection with different conditions of temperature and humidity, as reported by results in Table 2. Chun-Chieh and Chih-Shan (2006) reported the 90% and 99% inactivation of four different viruses, representative of ssDNA, ssRNA, dsDNA, and enveloped dsRNA categories and the ozone concentration required for the same inactivation level was lower at 85% RH than at 55%. The humidity is an important parameter to take into consideration because, under rather dry environmental conditions, the disinfection procedure could require considerably longer exposure times. In fact, microorganisms die more rapidly with increased humidity that favours the formation of radicals. Most of the studies reported in the literature have regarded the analysis of environmental conditions simulated in the laboratory in small chambers or cabinets with experimental conditions, in terms of temperature and relative humidity, set to room temperature and relative humidity >55%. Yano et al. 2020 described the inactivation of SARS-CoV-2 by gaseous ozone treatment using a concentration of 1.0 ppm ozone for 60 min and 6.0 ppm of ozone at 55 min at room temperature, 25°C, and relative humidity of 60–80%. An important reduction was obtained after exposure of 6.0 ppm ozone at 55 min, from 2.0*10^6^pfu/mL to 1.0*10^3^pfu/mL. Ozone gas was also effective against a pseudovirus at short contact times, below 30 min, as reported by Zucker et al. 2021 in their work. A recent study demonstrated also the efficacy of air treatment for phage and MNV-1 (eukaryotic murine norovirus) inactivation using low ozone concentrations, 1.13 ppm ± 0.26 ppm and 0.23 ppm ± 0.03 ppm, respectively, at various relative humidity levels and exposure times of up to 70 min. The inactivation of φX174, MS2 (phages) and MNV-1 was obtained with an exposure of 40 min at 85% relative humidity; while for other phages (PR772 and φ6) exposure for 10 min was enough [Dubuis et al. 2020].

Although in our working conditions, similar to daily use in healthcare facilities, we did not test the antiviral action on pathogenic viruses nor their surrogates, we analyzed the ozone efficacy on a selected gram-negative bacterium, used as an indicator of microbial contamination, in a real environment and we noticed no significant differences in bacterial inactivation between 35–55 % RH and a temperature range 16–25°C with an ozone concentration of 1.6 ppm for 70 min.

In Table 3, we report the results obtained in the air and on surfaces in a classroom that was used or not by students (ozonation was always conducted in the absence of people). In the presence of people, both air and surfaces were more contaminated before the ozone treatment and we observed a 90% microbial load reduction in the air (from 100 CFU/plate to 11 CFU/plate), while in the absence of people starting from a cleaner condition, the complete destruction of microorganisms was obtained. Microbiological analysis of the surfaces showed very good results, unregarding the presence of people. For these experiments, SAS instrument for air control was positioned in the middle of the classroom and three ozone-generators were positioned at the sides of the triangular room to guarantee the right concentration of ozone in all parts of the room and swabs were performed randomly on desks that had been previously used by the students to control the surfaces. Moreover surfaces were analysed also in the absence of students and normal environmental contamination was observed before sanification. In these cases, the environmental conditions were set to T=25°C and RH ranged between 45 and 55%. No differences were observed in ozone efficacy in these circumstances.

**Table 3:** Total microbial load estimated on sampling performed on different surfaces (CFU/plate) and in the air (CFU/m^3^) in a classroom (results are averaged on 10 measurements)

**AIR and surface analysed**	(PCA)* Pre-treatment	(PCA)* Post-treatment
Air	10 ± 1	-
Air with people	100 ± 4	11 ± 2
Surfaces	14 ± 1	3 ± 1
Surfaces with people	36 ± 1	2 ± 1

*Plate count agar

## Conclusions

Our study aims to define the efficacy and the optimization of ozone concentration achievable in real environments, such as offices and classrooms. These data could be used to lay the groundwork of sanification in defined ambients. In detail, this study provides an analysis of the microclimate influence on ozone efficacy in indoor environments. Different conditions of temperature, relative humidity, and distance from the ozone generator do not affect the reduction of microbial load and the commercial machine used provides a good diffusion of the gas during the sanitization operations. This permits the elimination and inactivation of microbial airborne species and also those that can be present on and under surfaces. The concentration of ozone was also evaluated after the sanitization process to ensure the total reduction of ozone at the end of the treatment, because of environmental/occupational hazard concerns. As already stated in the literature, the important factor for the inactivation of microorganisms is the total ozone dose which is calculated as the product of exposure time and concentration. According to literature data and close to the value suggested, our results pointed to the total dose of 112 min [ppm] for the sanification of the environments. These data can be used for reducing ozone concentration although assuring safe disinfection under different conditions.

## Data Availability

Not applicable
